# Supplementary material for: Detection and characterization of constitutive replication origins defined by DNA polymerase epsilon
Source: BMC Biol. 2023 Feb 24;21:41. doi: 10.1186/s12915-023-01527-z (PMC9960419; doi:10.1186/s12915-023-01527-z)
Supplement: Supplementary file 4 — Additional file 4: Fig. S1. Examples of mORI detection for locations described in the Shinbrot et al. 2014 article. Fig. S2. Distribution of distances between mORI positions compared to random genomic locations. Fig. S3. Distribution of mORI across chromosomes from the hg19 reference genome and number of mORI, with and without chromosome length standardization. Fig. S4. Overlap between various ORI detection methods. Fig. S5. Number of G-quadruplexes at specific distance from the replication origins, expressed as the fold change with respect to the minimum value from each row. Individual rows were obtained using various replication origin detection methods and results of the method concordance obtained using the MNVA algorithm. Fig. S6. Number of G- quadruplexes at specific distance from the replication origins, expressed as the fold change with respect to the minimum value from each row. The first row was obtained for all 65 329 positions from the core SNS-seq ORI set (Akerman et al. 2020), the following rows were obtained by randomly selecting a subset of 5 000 positions, repeated 10 times. Fig. S7. Number of topologically associating domain (TADs) regions (middle or border) at a given distance from the mORI replication origins, expressed as the fold change with respect to the minimum value from each row. Fig. S8.: Histogram of the PMA score obtained for peaks identified by the mORI detection algorithm for all samples combined. [file 12915_2023_1527_MOESM4_ESM.pdf]

Detection and characterization of constitutive replication origins defined by DNA polymerase epsilon

Roman Jaksik, David A. Wheeler, Marek Kimmel

Supplementary figures

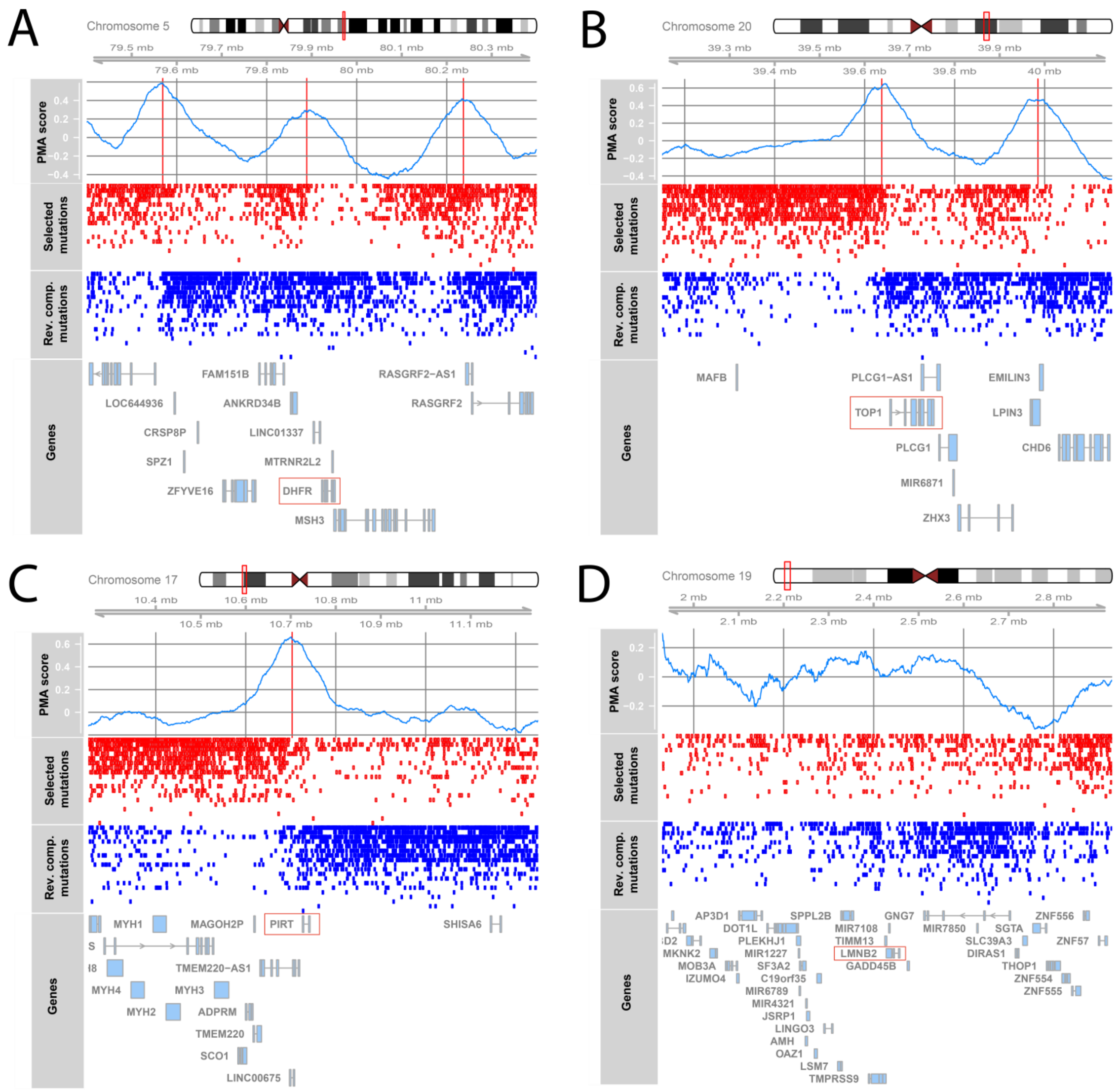

Fig. S1: Examples of mORI detection for locations described in the Shinbrot et al. 2014 article. Blue line shows the PMA score calculated based on combined POLE-exo mutated samples; red line indicates the position of detected mORI; blue and red squares mark the position of context dependent mutations characteristic to the PMA pattern with blue being reverse complementary to red; light blue squares mark the position of known human genes in the plotted region. **A**) ORI in the vicinity of the DFHR gene; **B**) ORI in the vicinity of the TOP1 gene; **C**) ORI in the vicinity of the PIRT gene; **D**) Vicinity of the LMNB2 gene (mORI was not detected)

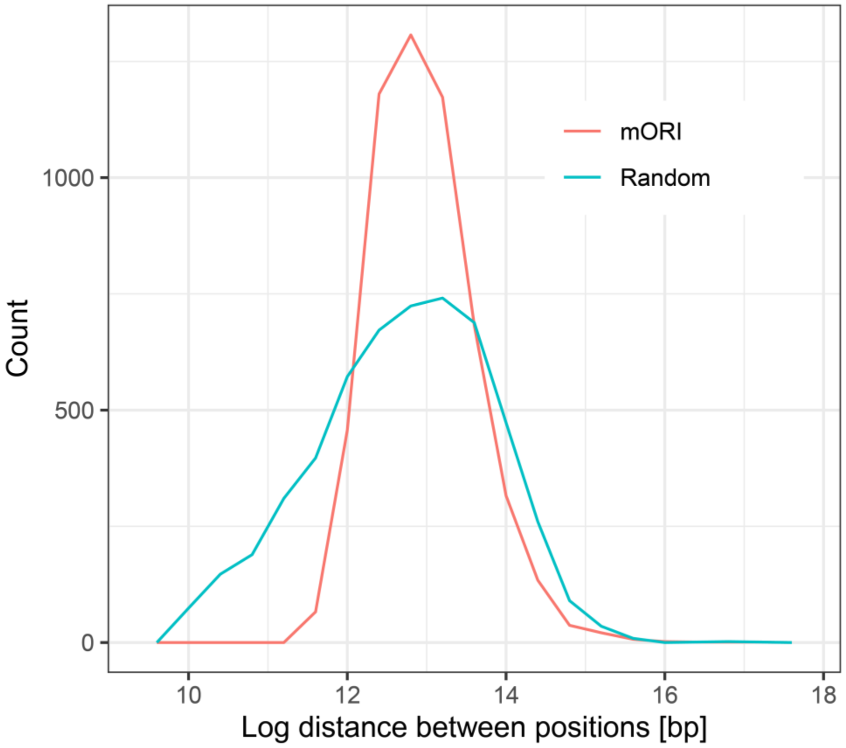

Fig. S2: Distribution of distances between mORI positions compared to random genomic locations.

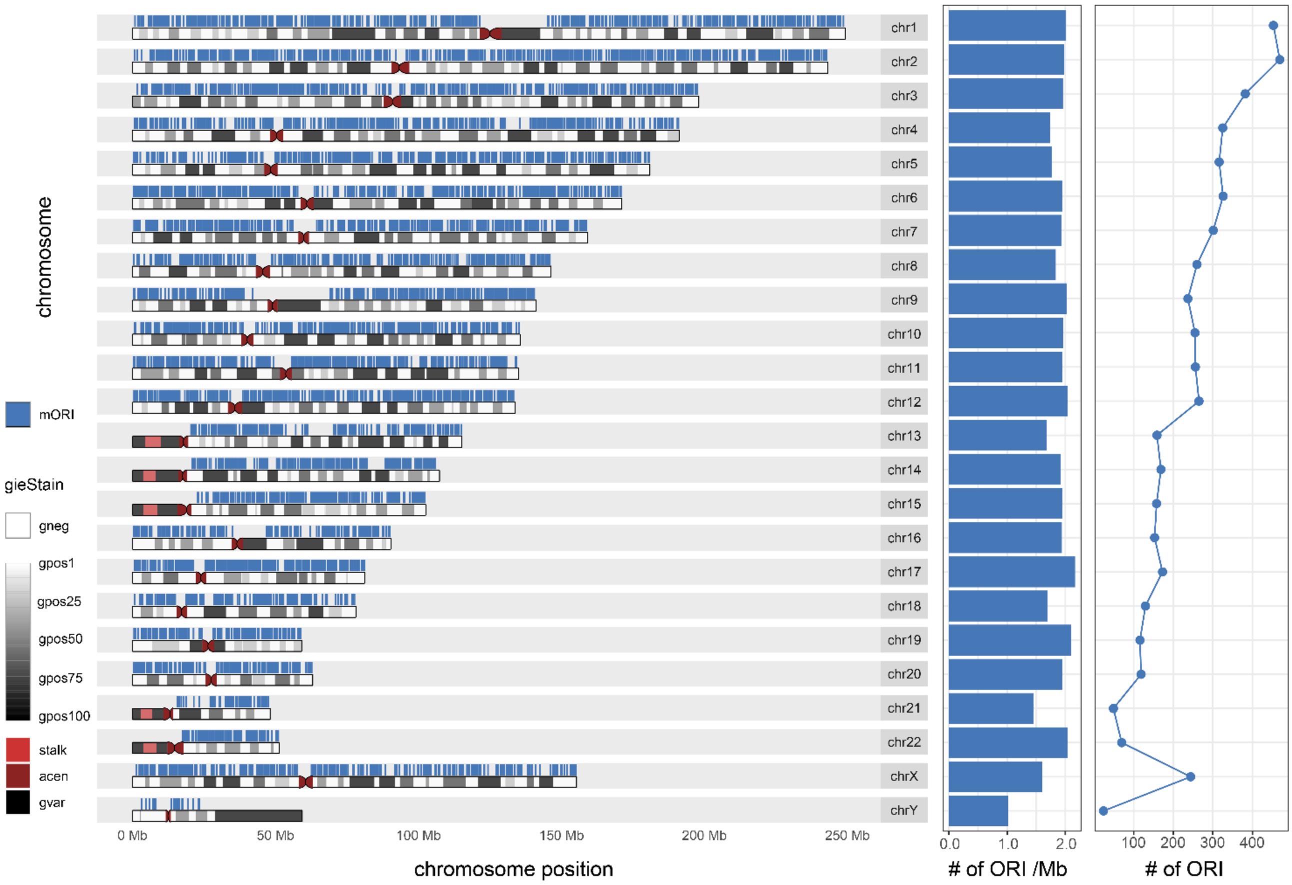

Fig. S3: Distribution of mORI across chromosomes from the hg19 reference genome and number of mORI, with and without chromosome length standardization

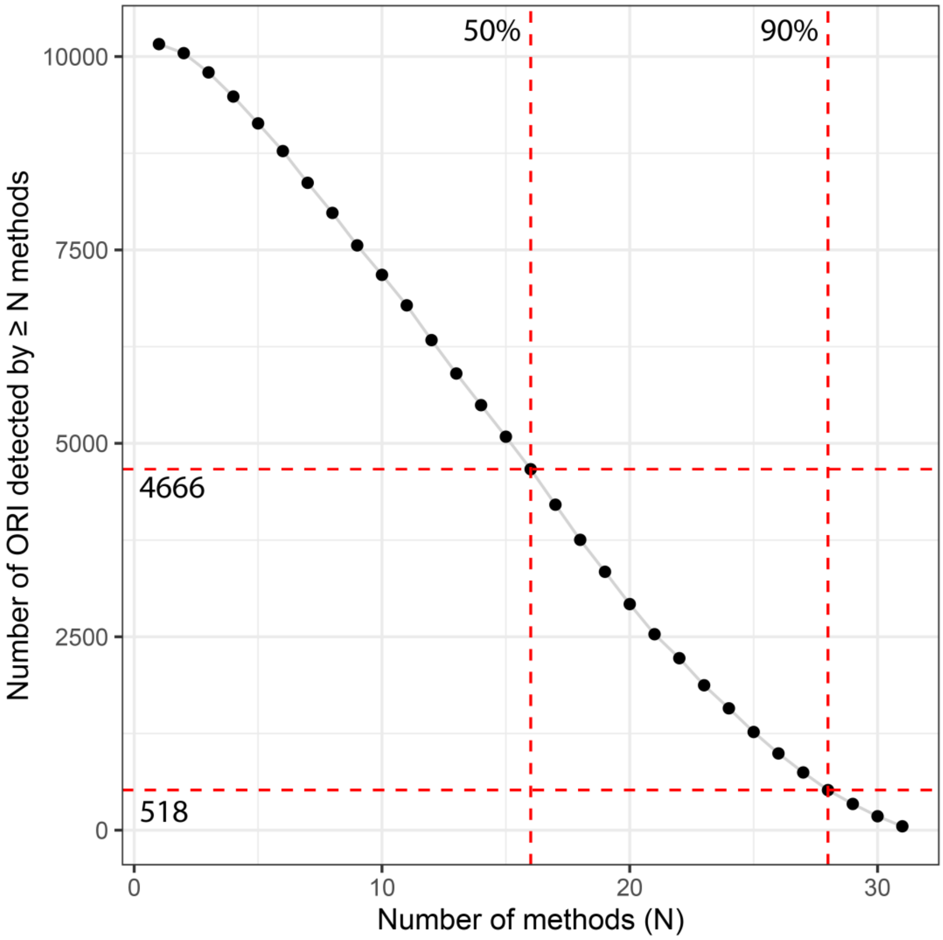

Fig. S4: Overlap between various ORI detection methods

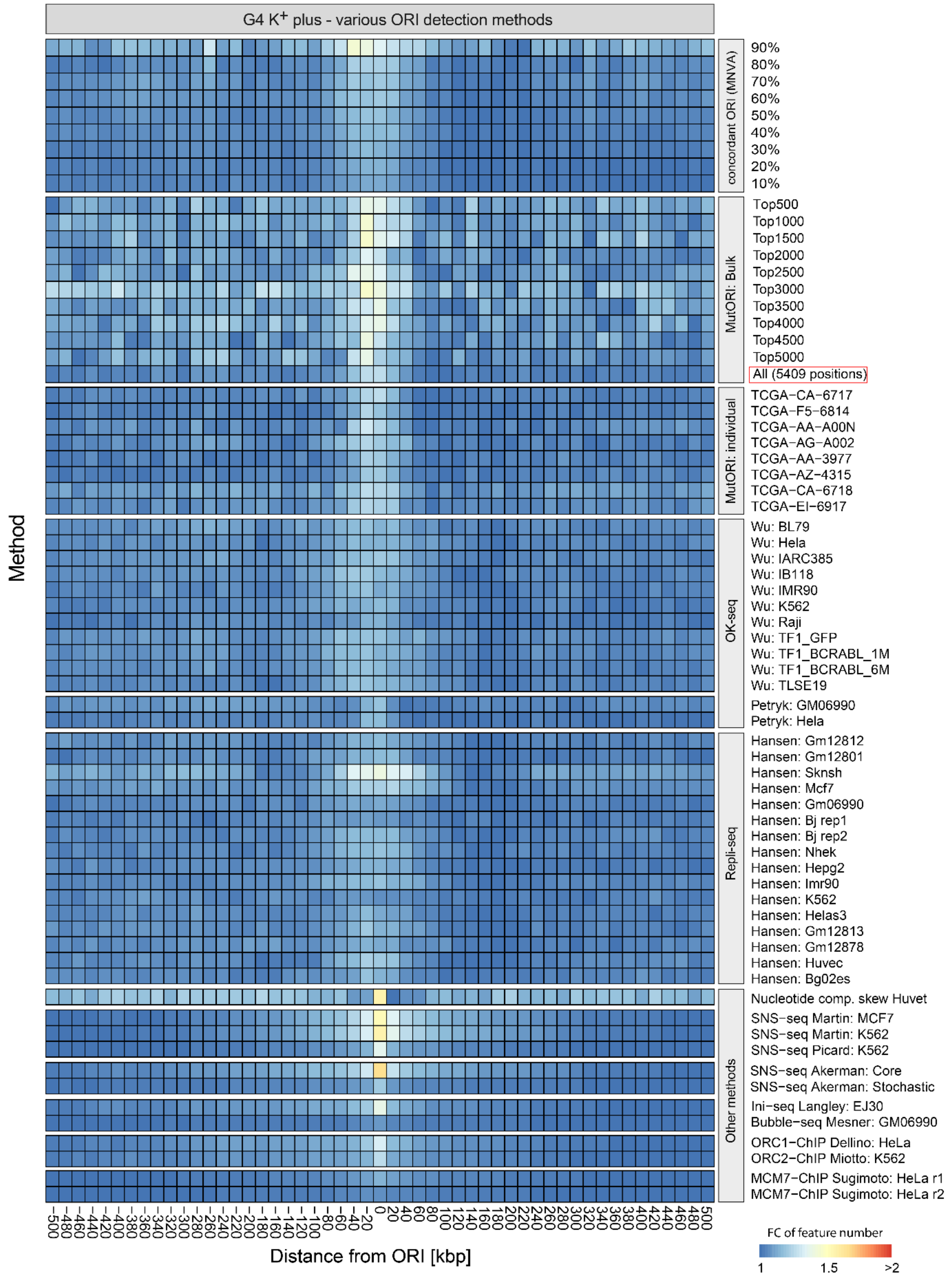

Fig. S5: Number of G-quadruplexes at specific distance from the replication origins, expressed as the fold change with respect to the minimum value from each row. Individual rows were obtained using various replication origin detection methods and results of the method concordance obtained using the MNVA algorithm.

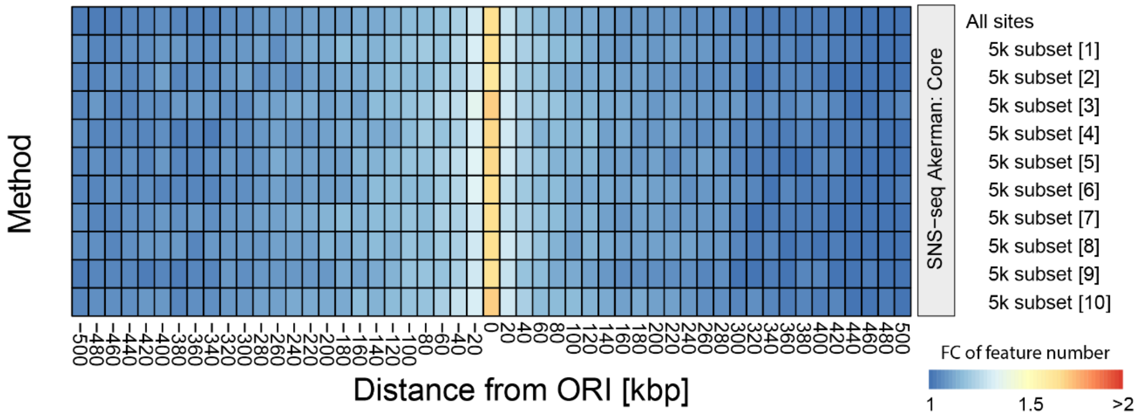

Fig. S6: Number of G- quadruplexes at specific distance from the replication origins, expressed as the fold change with respect to the minimum value from each row. The first row was obtained for all 65 329 positions from the core SNS-seq ORI set (Akerman et al. 2020), the following rows were obtained by randomly selecting a subset of 5 000 positions, repeated 10 times.

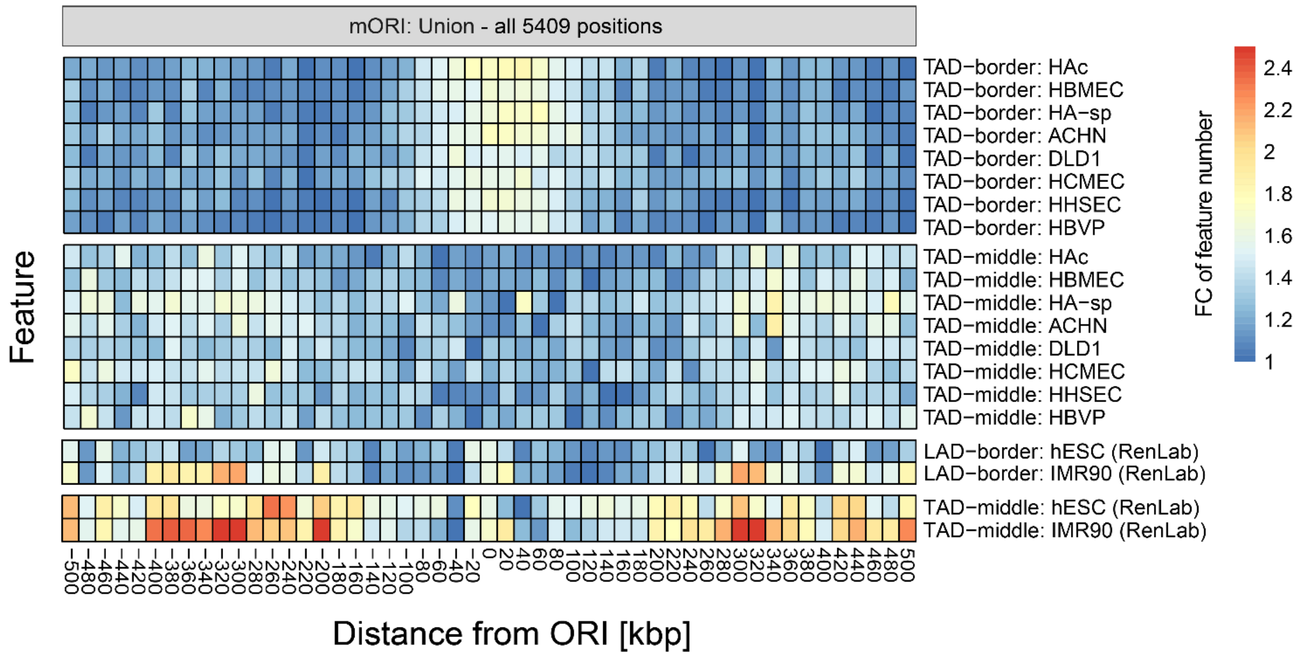

Fig. S7: Number of topologically associating domain (TADs) regions (middle or border) at a given distance from the mORI replication origins, expressed as the fold change with respect to the minimum value from each row.

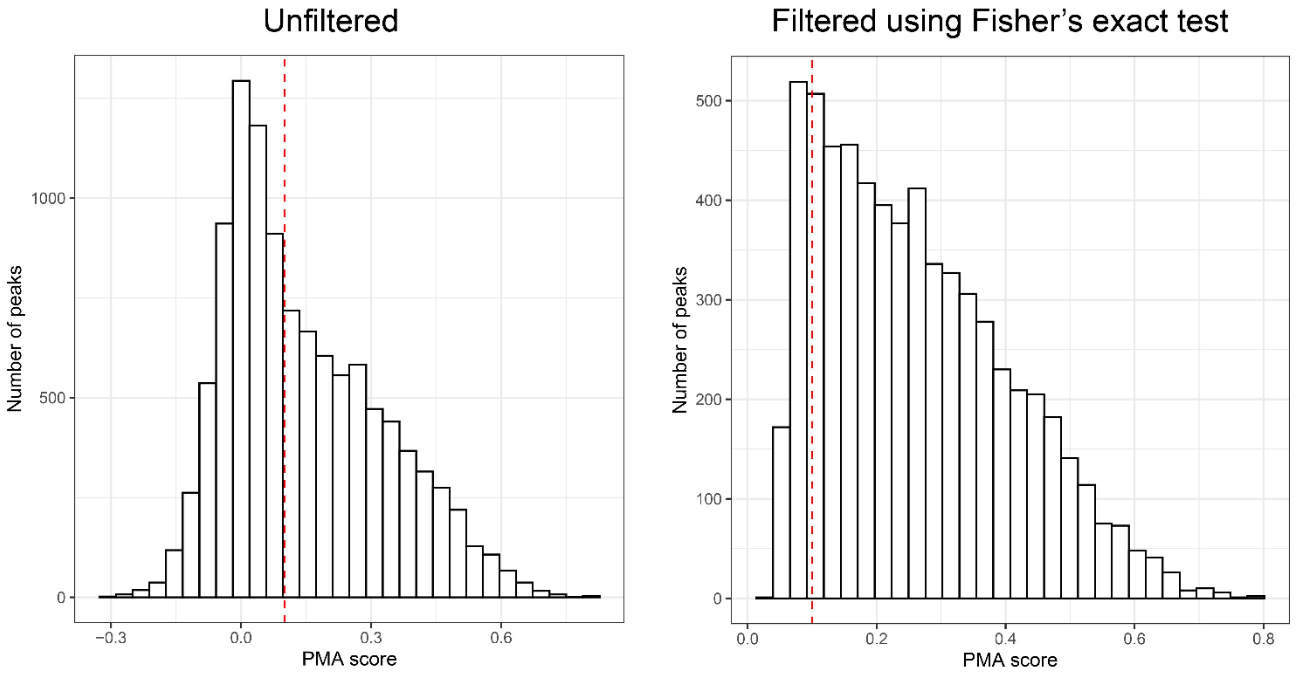

Fig. S8: Histogram of the PMA score obtained for peaks identified by the mORI detection algorithm for all samples combined. The selected cutoff = 0.1 is marked on the plot. The plot on the right was created after excluding certain positions based on Fisher's exact test (for details see materials and methods).
